# Effects of a 12-Week Diet versus Diet plus Aerobic and Resistance Exercise Program on Acylated and Desacylated Ghrelin, and Ghrelin O-Acyltransferase in Adolescent Girls with Obesity

**DOI:** 10.3390/ijerph19031480

**Published:** 2022-01-28

**Authors:** Hyun Jun Kim, Young Jin Tak, Sang Yeoup Lee, Jeong Pyo Seo

**Affiliations:** 1 Department of Physical Education, Kyungnam University, Changwon 51767, Korea; kimhj@kyungnam.ac.kr; 2 Biomedical Research Institute, Pusan National University Hospital, Busan 49241, Korea; 03141998@naver.com; 3 Department of Family Medicine, Pusan National University School of Medicine, Yangsan 50612, Korea; 4 Family Medicine Clinic, Biomedical Research Institute, Pusan National University Yangsan Hospital, Yangsan 50612, Korea; 5 Department of Medical Education, Pusan National University School of Medicine, Yangsan 50612, Korea; 6 Sehwa Girls’ High School, Changwon 51581, Korea; dzmong@naver.com

**Keywords:** acylated ghrelin, adolescent, deacylated ghrelin, exercise, ghrelin O-acyltransferase, obesity

## Abstract

This study investigated the effects of a 12-week diet versus diet plus aerobic and resistance exercise programme on acylated ghrelin (AG), desacylated ghrelin (DAG), and ghrelin O-acyltransferase (GOAT) concentrations in girls with obesity. We randomised 30 adolescents with obesity to a 12-week aerobic and resistance exercise group (EG) or a control group (CG). At baseline and at 4, 8, and 12 weeks, we measured their body composition, lipid profile, glucose, AG, DAG, and GOAT concentrations. In the EG, the body fat percentage decreased by 2.37% and was significantly lower than that in the CG. The DAG concentrations significantly increased by 48.3% and 27.4% in the EG and CG, respectively. At 4, 8, and 12 weeks, DAG concentrations were significantly higher in the EG than in the CG. AG concentrations were higher at week 12 than at baseline in both groups. In both groups, the GOAT concentrations increased at weeks 8 and 12; however, no between-group differences were observed in the changes in GOAT concentrations. This study showed increased DAG concentrations and non-significant changes in AG and GOAT concentrations after a 12-week aerobic and resistance exercise programme in girls with obesity. These findings suggest that an aerobic and resistance exercise programme influences appetite-regulating hormones, mainly through changes in DAG concentrations.

## 1. Introduction

Obesity in adolescence has become a global health issue, causing metabolic impairments and psychosocial distress [1]. The rate of spontaneous remission of obesity is low; at least 90% of adolescents with obesity are reported to remain overweight or obese in young adulthood [2]. The treatment of adolescent obesity includes long-term behavior modification: increasing physical activity and improving eating behaviors [3]. Fewer options for weight loss are available for adolescents since anti-obesity medication or bariatric surgery are limited to adults [1]. Additionally, considering that adolescents are in the phase of physical development, intensive dietary interventions, including caloric restriction, can be inappropriate for weight management [3]. Therefore, determining the effects of exercise on changes in the concentrations of appetite-related hormones is necessary to develop effective therapeutic approaches for adolescent obesity. However, the associations between exercise-induced energy expenditure and appetite have been studied mostly in adults, with little research conducted in adolescents [4]. Furthermore, related adolescent studies were short-term or assessed appetite using visual analogue scales [5,6].

Ghrelin, the only orexigenic hormone from the gut (primarily secreted by the gastric mucosa), is involved in glucose metabolism, food intake, and energy homeostasis. It exists in two forms: acylated (acyl ghrelin [AG]) and desacylated (des-acyl ghrelin [DAG]) [7]. Ghrelin O-acyltransferase (GOAT), identified in 2008, has been considered the main enzyme responsible for ghrelin acylation and is emerging as an important molecule of interest in the obesity management field [8]. DAG was once considered to be an inactive degradation product of the acylated form, but many studies have suggested that DAG performs distinctive activities through various physiological and pathophysiological pathways [7]. DAG supports or antagonizes the activities of AG and, depending on circumstances, induces metabolic effects completely independently of AG [8]. Central DAG administration stimulates feeding via activating orexin-expressing neurons, while peripheral DAG administration suppresses feeding in a ghrelin receptor-independent manner [9,10,11]. AG is the only known orexigenic hormone peripherally produced (mainly in the stomach) in humans. In contrast, DAG inhibits food intake and delays gastric emptying; thereby, inducing a state of negative energy balance and a decrease in body weight [12]. Although the results are contradictory, available data reveal that mice and humans with obesity have lower DAG concentrations than their normal-weight counterparts. In contrast, AG concentrations are similar regardless of obesity status, indicating that obesity may reflect a relative DAG deficiency and that DAG concentrations can be regulated by body weight [13,14]. Additionally, studies have shown that people with obesity and metabolic syndrome have a higher AG/DAG ratio [15] and that this ratio could be modulated by medical intervention [16]. Recently identified as the only known enzyme capable of the unique n-acyl modification of ghrelin, GOAT has emerged as an important molecule of interest in metabolic physiology. Substantial evidence has supported the involvement of GOAT in linking AG and DAG and, thus, regulating energy homeostasis [8]. Research has demonstrated that GOAT concentrations in humans depend on the metabolic environment, with increased concentrations in patients with obesity and decreased concentrations in patients with anorexia; this suggests that GOAT counteracts the adaptive changes of ghrelin observed under these conditions and ultimately contributes to the development or maintenance of anorexia and obesity [17]. 

Controversial results have been reported regarding ghrelin concentrations and exercise in humans. Some research showed no effects of short- or long-term exercise on circulating ghrelin concentrations [18,19], while other studies demonstrated increased ghrelin concentrations after long-term exercise [20,21]. Dorling et al. [18] reported that the modulating effects of adiposity, sex, and habitual physical activity on ghrelin response to acute and chronic exercise interventions were inconsistent. Prado et al. [19] found that personalized aerobic training three times a week (180 min/wk) for 24 weeks was efficient to reduce body weight and fat mass (%) in the obese group; nevertheless, no change in total ghrelin concentration were seen in obese girls, but a decrease in total ghrelin concentration was observed in obese boys after 24 weeks of therapy (*p* < 0.05). According to a recent systemic review, three long- (≥2 weeks) and very long-term (≥12 months) exercise training programmes resulted in increased total and DAG production along with weight loss in overweight or obese children and adolescents [22]. These discrepancies may be attributed to differences in study populations; duration and type of exercise; or methods of measuring ghrelin concentrations. To our knowledge, no research has yet been conducted regarding changes in GOAT after exercise or its role in exercise in adolescents. Therefore, with the availability of assays for measuring plasma DAG and GOAT concentrations [23], we aimed to prospectively investigate the effect of a 12-week diet versus diet plus aerobic and resistance exercise programme on AG and DAG concentrations in adolescents with obesity, and to examine whether GOAT plays a causal role in DAG elevation with no change in AG concentrations. We hypothesized that the increase in total ghrelin concentrations due to exercise might be attributed to DAG, not AG; further, GOAT concentrations would be affected by exercise, thereby causing changes in DAG and AG concentrations.

## 2. Materials and Methods

### 2.1. Trial Design

This randomised, placebo-controlled, clinical trial was approved by the Institutional Review Board at Busan National University Yangsan Hospital (IRB No. 04-2015-015). The study was performed in accordance with the principles of the Declaration of Helsinki and Korea Good Clinical Practice between 1 August 2015, and 31 December 2015. Written informed consent was obtained from all of the participants before enrolment. This trial is registered with ClinicalTrials.gov (Identifier: NCT04447391). 

### 2.2. Study Participants

According to the National School Health Examination data in Korea, the prevalence of obesity in children and adolescents aged 6–18 has increased by 1.7 times over the past 10 years. Accordingly, each school is encouraged to operate a school-based obesity prevention programme [24,25,26]. Our author’s school has implemented it and was scheduled to implement it this period. Therefore, the candidates were recruited from a girls’ high school in Changwon, Korea. The eligible participants included girls aged between 16 and 18 years who were classified as having overweight or obesity, defined as the 85th percentile of body mass index (BMI) or above based on the 2007 growth chart for Korean children aged 2–18 years [27]. The exclusion criteria included the following: participation in any weight-management programme in the past 6 months; engagement in exercise programmes other than the regular physical education sessions at school; current smoker status; consecutive amenorrhea for ≥2 months; treatment for hereditary diseases, mental disorders, hypertension, diabetes mellitus, or musculoskeletal impairments; and use of medication or supplements for weight control.

### 2.3. Randomisation and Follow-Up

**Figure 1:**
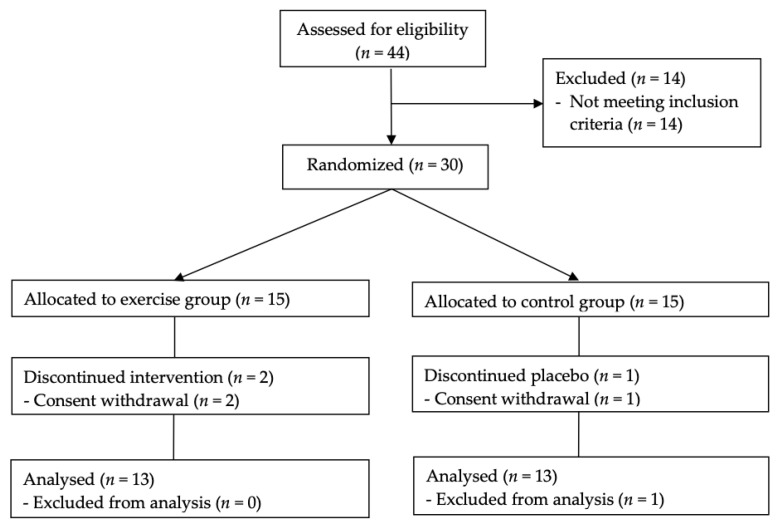
Flow diagram of the study selection process. Of the 44 enrolled candidates, 30 participants were randomised in a 1:1 ratio to either the exercise group or control group.

Overall, 44 girls were screened, 14 patients were excluded because they did not meet the inclusion criteria, and 30 were finally enrolled. After baseline measurements were taken, they were randomly assigned to one of the following groups through block randomisation using randomised numbers. They were provided with identification numbers upon recruitment: exercise group (EG) (*n* = 15), a 12-week aerobic and resistance exercise programme; or control group (CG) (*n* = 15), no additional exercise. Randomisation codes were created by an expert in statistics using SAS version 9.3. If compliance to the exercise programme was <80%, the participant was considered to have dropped out of the study. Three participants withdrew from the study after 4 weeks (two EG; one CG); one participant in the CG declined to undergo the 12-week follow-up; thus, data were available for 26 (86.7%) participants at 12 weeks (Figure 1).

### 2.4. Interventions

At baseline, the participants were educated on healthy dietary habits through one-on-one consultation. At the study initiation, the participants were requested to reduce their calorie intake by 300 kcal/day compared to the baseline calorie intake; however, to have at least 1500 kcal/day. Five days a week, the participants had 200 fewer calories a meal at school lunches than usual.

Girls in the EG participated in the aerobic and resistance exercise programme, consisting of a warm-up, aerobic exercise, resistance training, and a cool-down, 3 days a week (Mondays, Wednesdays, and Fridays) after school for 50 min from 17:30, under the supervision of a trainer. After 5 min of warm-up, 20 min of walking was performed for aerobic exercise, beginning at a low intensity (maximum heart rate, 55–64%; or rating of perceived exertion [RPE], 11–12) [28] during weeks 1–4; and then at a moderate intensity (maximum heart rate, 65–75%; or RPE 13–15) during weeks 5–12, as based on the Karvonen formula [29]. The heart rate was measured using an X-trainer heart rate monitor (Polar Electro Oy, Kempele, Finland). For the resistance training, rubber band exercises were performed for 20 min; these consisted of nine movements to exercise the large and small muscle groups: bench press, squat, elbow curl, seated row, knee curl, sit-up, knee extension, overhead press, and seated leg press. Three sets of 8–10 repetitions for each movement were performed at an RPE of 11–12 for the first four weeks; 3 sets of 10-15 repetitions were performed at the same RPE during weeks 5–12. The type of rubber band was chosen such that the participant could perform 10 repetitions without failure at the speed of 10–15 times/min. Each session was wrapped up with 5 min of stretching and light running. 

### 2.5. Baseline and Follow-Up Measurements

Waist circumference (WC) was measured using a tape placed at the thinnest area between the lowest part of the ribs and the iliac crest of the pelvis during normal exhalation. Body weight and height were checked using a digital scale, with the examinee wearing a light gown but no shoes. The BMI was calculated as body mass (kg) divided by height squared (m^2^). Body composition, including body fat and lean muscle mass (LBM), was checked via bioelectric impedance analysis (Inbody 370, Biospace Co. Ltd., Seoul, Korea). Blood tests for the measurement of plasma glucose, lipid, AG, DAG, and GOAT concentrations were conducted at baseline (pre-randomisation) and at 4, 8, and 12 weeks after randomisation. Blood samples were obtained using vacutainer tubes (BD Vacutainer® Plus plastic K2 EDTA tubes) between 8:00 a.m. and 9:00 a.m. from the antecubital vein after a 12-h fast; the samples were then immediately centrifuged at 4 °C and stored at −70 °C until testing. Plasma concentrations of glucose, triglycerides, high-density lipoprotein cholesterol, and low-density lipoprotein cholesterol were measured using a biochemical analyzer (BTS370, BioSystmes, Co., Barcelona, Spain). AG, DAG, and GOAT concentrations were measured using a commercial ELISA kit (Human Acylated ghrelin (AG) ELISA Kit, Human Desacyl Ghrelin ELISA Kit, and Human Ghrelin O-Acyltransferase ELISA Kit, MyBioSource, San Diego, CA 92195-3308, USA). The inter- and intra-assay coefficients of variation (CV) were 7.0% and 5.2%, respectively, for AG; 8.6% and 6.5%, respectively, for DAG; and 7.6% and 5.8%, respectively, for GOAT.

### 2.6. Sample Size

The sample size was calculated based on the results of our previous study [12]. The estimated sample size was 13 participants per group for 80% power to detect a 20% difference in changes in DAG concentrations from baseline between the groups, assuming a standard deviation of 15% and an alpha error of 5%. In total, 30 participants (15 per group) were required, with an assumed dropout rate of 20%.

### 2.7. Statistical Analyses

Per-protocol analyses were performed. The normality assumption was tested using Shapiro–Wilk’s test. Data are expressed as means ± standard deviations or medians (interquartile ranges). Intergroup comparisons of baseline characteristics were performed using a two-sample *t*-test (after log transformation for skewed data) for parametric variables or Mann–Whitney’s U test for non-parametric variables (even after log transformation). To verify the differences in changes between baseline and weeks 4, 8, and 12, a repeated measures analysis of variance (ANOVA) was performed for the percentage body fat and the AG, DAG, and GOAT concentrations; the Friedman ANOVA was used for the AG/DAG ratio. A *p* value ˂ 0.05 was considered statistically significant. SPSS version 21.0 (IBM Inc., Armonk, NY, USA) was employed for analysis.

## 3. Results

### 3.1. Baseline Characteristics of the Participants

A comparison of the baseline characteristics between the groups is listed in Table 1. No significant differences in baseline BMI, WC, and body composition were observed (Table 1). Furthermore, there were no significant differences in fasting glucose concentrations and lipid profile at baseline between the groups. However, DAG concentrations were higher in the EG than in the CG (381.0 ± 247.2 pg/mL vs. 717.8 ± 59.8 pg/mL) (Table 1).

### 3.2. Changes in Body Composition, Glucose Concentrations, and Lipid Profile

**Figure 2:**
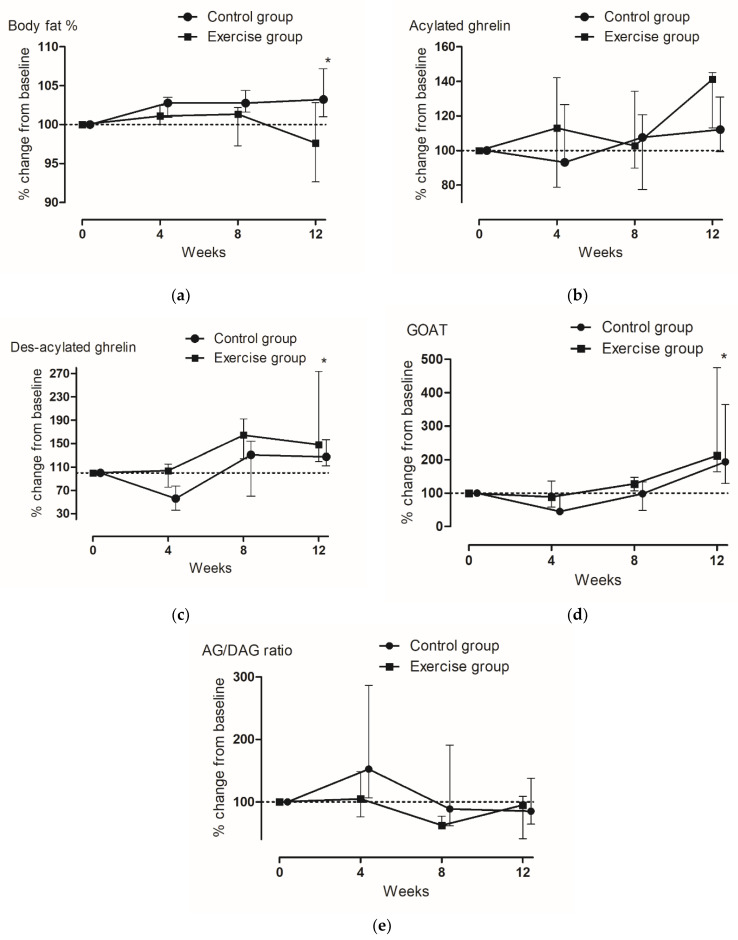
Changes in the percentage body fat and metabolic hormone concentrations from baseline in girls with obesity. (**a**) Body fat (%), (**b**) aclylated ghrelin, (**c**) des-acylated ghrelin, (**d**) GOAT, (**e**) AG/DAG ratio. Data are expressed as medians with interquartile ranges (I bars). Asterisks indicate significant between-group differences over time (*p* < 0.05). *p*-values were calculated by repeated measures ANOVA. AG, acylated ghrelin; DAG, desacylated ghrelin; GOAT, ghrelin O-acyltransferase.

At week 12, no significant absolute values of changes in body composition and concentrations of glucose and lipid profile were observed between the groups (Table 1). In terms of the percentage changes from baseline, body fat decreased by 2.37% at week 12 in the EG, while in the CG, body fat slightly increased by 3.23% (Figure 2a). At weeks 4, 8, and 12, the percentage body fat was significantly lower in the EG than in the CG (Figure 2a). There were no significant changes in BMI, WC, and muscle mass over time in either group (Table 2). 

### 3.3. Changes in Gut Hormones

Compared to those at baseline, AG and GOAT concentrations significantly increased at week 12 in both groups (Table 3). However, the difference in the increases in AG and GOAT concentrations between the groups was not significant. In terms of the percentage change from baseline, GOAT concentrations decreased at week 4 in both groups and increased significantly thereafter at weeks 8 and 12; the increases were greater in the EG than in the CG (112.1% vs. 93.7%) (Figure 2d). Similarly, by week 12, DAG concentrations significantly increased by 48.3% and 27.4% compared to the baseline values in the EG and CG, respectively; at weeks 4, 8, and 12, the increases in DAG concentrations were significantly higher in the EG than in the CG (Figure 2c). Contrastingly, AG concentrations were higher at week 12 than at baseline in both of the groups (41.2% vs. 12.1%); however, there was no significant difference in the change in AG concentrations from baseline to week 12 between the groups (Figure 2b). At week 12, no significant absolute values and percentage change of the AG/DAG ratio were observed between the groups.

## 4. Discussion

Exercise is most frequently performed to increase energy expenditure, creating a sustained energy deficit. However, compensatory responses resulting from weight loss can undermine the efficacy of exercise for theoretical weight loss. Previous studies have reported inconsistent results regarding the effect of exercise on changes in body composition and appetite-related hormones [4]. Moreover, many of the studies included adults, and, as far as we know, no researchers have specifically measured GOAT in adolescents [30]. We aimed to determine whether a 12-week aerobic and resistance exercise programme would alter AG, DAG, and GOAT concentrations in adolescents with obesity. Participation in the aerobic and resistance exercise programme resulted in a significant decrease in body fat and significant increase in plasma DAG concentrations. These findings suggest that long-term exercise can help reduce body fat by decreasing appetite or at least not triggering food intake.

Our previous study on children with obesity found that a 12-week aerobic and resistance exercise programme resulted in increases in plasma total ghrelin and DAG concentrations but not in AG concentrations. This increase was greater in the EG than in the CG [12]. However, DAG concentrations were not measured directly in that study and were determined by a simple equation (total ghrelin minus AG). Moreover, since methods for measuring GOAT concentrations were not available at that time, the possible physiological effects of GOAT on changes in DAG and AG concentrations during exercise remained to be elucidated. To overcome these limitations, this study directly measured changes in AG, DAG, and GOAT concentrations during a 12-week aerobic and resistance exercise programme. The increase in DAG concentrations was greater in the EG than in the CG. The rise in DAG in CG is presumed to be due to dietary restriction rather than not exercising. Previous studies also showed that dietary restriction increased DAG in overweight children and obese adults [12,31]. However, there were no between-group differences in absolute values of GOAT and AG concentration changes. These novel data suggest that aerobic and resistance exercise may influence DAG concentrations through a different pathway. Considering previous studies that other enzymes, including acyl protein thioesterase 1 and lysophospholipase I, play an important role in determining the concentration of DAG [32,33], it is necessary to investigate including the changes in these enzymes during exercise programmes in the future. 

A recent study showed that concentrations of AG and DAG (both fasting and prandial) and the AG/DAG ratio did not change between baseline and 6-12 months following different types of bariatric surgery in participants with obesity [34]. Similarly, no significant absolute values and percentage change of the AG/DAG ratio were observed between groups for 12 weeks in our study. Among the few studies including adolescents, one compared the acute effects of a 5-day aerobic exercise programme on AG and DAG concentrations and appetite, between adolescents with obesity and their normal-weight counterparts [6]. Consistent with our results, they reported that exercise caused an increase in DAG concentrations in the obesity group but a decrease in the normal-weight group. They also found that AG concentrations significantly increased after exercise. This increase was greater in normal-weight subjects than in subjects with obesity, indicating that exercise differentially affects AG concentrations depending on body weight. Based on the result that AG elevation post-exercise was concomitant with an enhanced subjective feeling of appetite, they speculated that these exercise-induced hormonal changes would limit the effectiveness of existing interventions, including exercise-based weight loss programmes for the treatment of obesity. This is in contrast with our previous study, which reported increased DAG concentrations and unchanged AG concentrations after a 12-week aerobic and resistance exercise programme in children with overweight, providing evidence of the favorable effects of exercise in improving energy metabolism in terms of DAG concentrations [12]. However, neither study measured DAG separately; instead, DAG concentrations were calculated by subtracting AG values from the total ghrelin values. Additionally, Kerry’s study [6] had a short duration (5 days), and only aerobic exercise was performed. However, it is clinically important to examine the effects of long-term exercise on body composition and appetite-related hormonal changes, since losing weight by either reducing calorie intake or increasing physical activity takes time and long-term efforts. The limitations of these previous studies prompted us to conduct the current trial to investigate the effect of long-term exercise in adolescents with obesity. Similar to that in our previous study, a 12-week aerobic and resistance exercise programme decreased body fat and increased DAG concentrations in adolescents with obesity. In our study, the body fat percentage decreased in EG, and the increase in DAG was greater in the EG than in the CG at week 12. It is presumed that DAG had a fat reduction effect along with the exercise effect. Although much remains to be revealed about the ghrelin-GOAT system, white adipose tissues weighed about 25–60% less in transgenic mice overexpressing DAG compared with the wild-type mice, with no change in brown fat [35,36]. Contrary to AG, administration of DAG suppresses genes involved in adipogenesis and lipogenesis, and then decreases lipogenesis, particularly in white adipose tissues [37]. There were no significant differences in absolute values of the changes in AG and GOAT concentrations before and after the trial between the EG and CG. Additionally, our observations are consistent with those of previous studies that demonstrated favorable effects of long-term exercise on body composition and some metabolic hormones/peptides in pediatric populations [38,39]. 

Our study had some limitations, including its relatively small sample size and the inclusion of only girls. As we did not directly monitor changes in energy intake and the subjective markers of appetite during the trial, whether the observed changes in DAG concentrations translate into actual changes in energy intake or appetite remains to be ascertained. In both EG and CG, similar alterations in AG were observed (Table 3). At baseline, the CG, as well as the EG, were educated on healthy dietary habits through one-on-one consultation and were requested to decrease their calorie intake. The authors guess this advice would have affected calorie consumption, resulting in a similar pattern of changes in the concentrations of metabolic hormones in both groups. Korean high schools provide lunch; therefore, all participants were provided with a fixed calorie-restricted diet, 5 days a week, at school lunchtime. In addition, although there were no differences in sociodemographic and anthropometric variables at baseline, the total ghrelin and DAG concentrations were different between the EG and CG. Therefore, as in our previous study [12,40], we compared the difference in the percentage change from baseline of hormone concentrations between the two groups. This is because there is a wide range of ghrelin concentrations among healthy individuals [41]. Further studies could investigate the possible reasons of the difference in DAG concentrations in girls with obesity. 

Despite the above limitations, to our knowledge, this is the first randomised controlled trial reporting increased DAG concentrations and unchanged AG and GOAT concentrations in adolescents with obesity who participated in a 12-week aerobic and resistance exercise programme. Moreover, we used a new direct ELISA assay [14] for the separate measurement of plasma AG, DAG, and GOAT concentrations so that we could further determine the effect of GOAT on the counter-regulation between AG and DAG.

## 5. Conclusions

Our results suggest that weight loss after a 12-week aerobic and resistance exercise programme was associated with increased DAG concentrations and unchanged AG concentrations. Considering its numerous health benefits, emphasizing the importance of exercise is necessary for improving general life quality, not just for the purpose of weight reduction, even though it could increase appetite depending on the individual. In this context, our novel findings may benefit those who are reluctant to start exercising because of concerns that exercise might trigger appetite and lead to an increase in food intake. Our results also imply that weight loss from long-term exercise could play a role in physiological mechanisms regulating energy balance through DAG rather than through AG or GOAT in adolescents with obesity.

## Figures and Tables

**Table 1:** Baseline characteristics and metabolic parameters of the participants.

Parameters	Exercise Group (*n* = 13)	Control Group (*n* = 13)	*p*
Age			
17	12 (92.3)	8 (61.5)	
18	1 (0.7)	5 (38.5)	
Weight (kg)	75.1 ± 8.2	78.0 ± 13.5	0.513
BMI (kg/m^2^)	27.1 (26.4–30.0)	29.7 (26.3–33.4)	0.397 *
WC (cm)	85.0 ± 5.5	88.5 ± 9.0	0.236
Body fat (%)	33.9 ± 2.7	35.5 ± 3.5	0.189
Lean body mass (kg)	45.3 ± 3.4	45.3 ± 5.7	0.964
TC (mg/dL)	155.7 ± 45.2	131.9 ± 15.6	0.086
Triglyceride (mg/dL)	55.0 (45.0–87.0)	52.0 (45.0–77.3)	0.652 *
HDL-C (mg/dL)	61.6 ± 15.4	57.0 ± 10.6	0.382
LDL-C (mg/dL)	79.9 ± 41.1	61.8 ± 14.7	0.148
Glucose (mg/dL)	88.0 (84.5–90.3)	87.0 (76.8–91.5)	0.567
Total ghrelin (pg/mL)	445.9 ± 236.0	799.5 ± 213.4	0.001
AG (pg/mL)	64.9 ± 16.5	81.7 ± 16.5	0.188
DAG (pg/mL)	381.0 ± 247.2	717.8 ± 59.8	0.001
AG/DAG ratio	0.27 ± 0.18	0.13 ± 0.09	0.002
GOAT (ng/mL)	9.3 (6.9–23.1)	15.0 (9.5–22.5)	0.535

Values are expressed as the means ± standard deviations (SD) or medians (interquartile ranges). BMI, body mass index; WC, waist circumference; TC, total cholesterol; HDL-C, high-density lipoprotein cholesterol; LDL-C, low-density lipoprotein cholesterol; AG, acylated ghrelin; DAG, desacylated ghrelin; GOAT, ghrelin O-acyltransferase; *p* value, by two-sample *t*-test for parametric variables, or * Mann–Whitney’s U test for non-parametric variables.

**Table 2:** Changes in anthropometric parameters of the two groups.

Parameters		Baseline	4 Weeks	8 Weeks	12 Weeks	Within-Group	Between-Group	Group × Time
Weight (kg)	EG	75.1 ± 8.2	75.2 ± 7.9	75.1 ± 8.3	74.3 ± 8.5	0.538	0.412	0.114
	CG	78.0 ± 13.5	78.5 ± 13.1	78.8 ± 13.6	79.2 ± 14.1			
BMI (kg/m^2^)	EG	27.1 (26.4–30.0)	27.2 (26.5–29.4)	27.1 (26.2–29.5)	27.1 (25.9–29.5)	0.542	0.163	0.097
	CG	29.7 (26.3–33.4)	28.9 (26.7–33.5)	29.9 (26.4–33.5)	30.0 (26.1–34.0)			
WC (cm)	EG	85.0 ± 5.5	83.4 ± 6.7	83.7 ± 6.6	84.2 ± 7.5	0.345	0.259	0.543
	CG	88.5 ± 9.0	88.0 ± 10.7	87.4 ± 8.2	86.5 ± 10.4			
BF (%)	EG	33.9 ± 2.7	34.1 ± 2.6	33.1 ± 2.6	33.3 ± 2.9	0.042	0.064	0.051
	CG	35.5 ± 3.5	36.4 ± 3.4	36.4 ± 3.4	36.3 ± 3.7			
LBM (kg)	EG	45.3 ± 3.4	45.1 ± 3.2	45.3 ± 3.4	45.2 ± 3.4	0.473	0.936	0.581
	CG	45.3 ± 5.7	45.1 ± 5.3	45.4 ± 5.8	45.5 ± 5.8			

Values are expressed as the means ± SD or medians (interquartile ranges). EG, exercise group; CG, control group; BMI, body mass index; WC, waist circumference; BF, body fat; LBM, lean body mass. *p* value obtained by a repeated measures ANOVA.

**Table 3:** Changes in the metabolic parameters of the two groups.

Parameters		Baseline	4 Weeks	8 Weeks	12 Weeks	Within-Group	Between-Group	Group × Time
TC (mg/dL)	EG	155.7 ± 45.2	139.4 ± 28.3	138.8 ± 32.9	161.2 ± 31.9	0.002	0.184	0.107
	CG	131.9 ± 15.6	129.6 ± 12.1	138.2 ± 19.1	144.5 ± 24.9			
Tryglyceride (mg/dL)	EG	61.6 ± 15.4	48.9 ± 11.3	54.2 ± 14.6	55.2 ± 13.0	0.001	0.985	0.193
	CG	57.0 ± 10.6	51.8 ± 10.4	53.4 ± 11.4	57.5 ± 7.7			
HDL-C (mg/dL)	EG	55.0 (45.0–87.0)	45.0 (45.0–83.0)	69.0 (51.8–93.0)	80.0 (64.8–115.3)	0.048	0.258	0.527
	CG	52.0 (45.0–77.3)	57.0 (45.0–71.8)	64.0 (49.5–82.8)	65.0 (48.8–95.5)			
LDL-C (mg/dL)	EG	79.9 ± 41.1	76.0 ± 27.1	68.9 ± 33.1	86.4 ± 27.5	0.198	0.253	0.162
	CG	61.8 ± 14.7	67.3 ± 11.2	70.8 ± 18.2	71.6 ± 21.7			
Glucose (mg/dL)	EG	88.0 (84.5–90.3)	82.0 (79.8–85.3)	88.0 (77.8–89.0)	85.0 (83.0–100.8)	0.236	0.187	0.026
	CG	87.0 (76.8–91.5)	81.0 (80.0–84.5)	85.0 (81.0–96.3)	79.0 (78.0–83.3)			
TG (pg/mL)	EG	445.9 ± 247.2	449.4 ± 214.6	654.8 ± 277.8	737.0 ± 296.1	0.088	0.042	0.392
	CG	799.5 ± 213.4	543.0 ± 342.4	1296.7 ± 539.1	1022.0 ± 298.1			
AG (pg/mL)	EG	64.9 ± 16.5	74.3 ± 32.3	76.4 ± 32.4	83.9 ± 34.7	0.017	0.372	0.707
	CG	81.7 ± 16.5	84.5 ± 16.5	81.7 ± 16.5	94.4 ± 16.5			
DAG (pg/mL)	EG	381.0 ± 247.2	375.1 ± 214.6	578.3 ± 277.8	653.0 ± 297.1	0.092	0.048	0.386
	CG	717.8 ± 215.8	458.5 ± 326.3	1214.0 ± 465.0	927.5 ± 299.1			
AG/DAG ratio	EG	0.27 ± 0.18	0.29 ± 0.21	0.18 ± 0.12	0.18 ± 0.13	<0.001	0.902	0.915
	CG	0.13 ± 0.09	0.24 ± 0.13	0.12 ± 0.07	0.11 ± 0.07			
GOAT (ng/mL)	EG	9.3 (6.7–23.8)	8.1 (6.0–14.3)	27.7 (8.3–35.7)	44.8 (16.1–53.8)	<0.001	0.096	0.220
	CG	15.0 (9.0–25.7)	7.9 (6.3–15.2)	20.1 (8.5–23.6)	43.5 (26.2–46.2)			

Values are expressed as the means ± SD or medians (interquartile ranges). TC, total cholesterol; HDL-C, high-density lipoprotein cholesterol; LDL-C, low-density lipoprotein cholesterol; TG, total ghrelin; AG, acylated ghrelin; DAG, desacylated ghrelin; GOAT, ghrelin O-acyltransferase. *p* value obtained by a repeated measures ANOVA.

## Data Availability

The data supporting the findings of this study are available on request from the corresponding author. The data are not publicly available due to their containing information that could compromise the privacy of the research participants.
